# Measurement properties of outcome measurement instruments for pediatric dysfunctional breathing: a systematic review

**DOI:** 10.1007/s11136-025-04078-8

**Published:** 2025-10-10

**Authors:** Vikram Mohan, Chandrasekar Rathinam, Derick Yates, Andra Voicu, Charanjet Malotra, Isabelle Riley, Olalekan Lee Aiyegbusi, Prasad Nagakumar

**Affiliations:** 1 Department of Rehabilitation and Sports Sciences, Faculty of Health and Social Sciences, Bournemouth University, Bournemouth, UK; 2 Birmingham Women’s and Children’s NHS Foundation Trust, Birmingham, UK; 3 Centre of Patient-Reported Outcome Research (CPROR), University of Birmingham, Edgbaston, Birmingham, UK

**Keywords:** Dysfunctional breathing, Asthma, Questionnaire, Validity, Reliability

## Abstract

**Purpose:**

Clinicians commonly use various outcome measurement instruments (OMIs) to assess dysfunctional breathing (DB) in children. However, no review has examined their psychometric properties. This article systematically reviewed the psychometric properties of OMIs in pediatric DB.

**Methods:**

Articles that developed or evaluated measurement properties of OMIs for pediatric DB were included. EbscoHost CINAHL Ultimate, Cochrane Library, Ovid Embase, EMCare, and Medline were searched from inception to October 10, 2024. Methodological quality and psychometric properties were assessed and synthesised using the COSMIN (COnsensus-based Standards for the selection of health Measurement INstruments) methodology.

**Results:**

After screening 14,240 references, three articles met the inclusion criteria, identifying two OMIs: the Hyperventilation Syndrome Ambroise-Paré (SHAPE) in French and the Nijmegen Questionnaire (NQ). SHAPE showed ‘*doubtful*’ OMI development and content validity with an ‘indeterminate’ rating for the results. One study reported *‘inadequate’* methodological quality for structural validity, though the property itself was rated ‘sufficient’. Another study had *‘very good’* methodological quality, with *‘sufficient’* ratings for the criterion and discriminative validity. The NQ was evaluated for convergent validity; the methodological quality was ‘*inadequate*’, but the property was rated ‘*sufficient*’. GRADE quality of evidence for SHAPE’s development and content validity was not graded due to indeterminate results. For the remaining measurement properties, evidence quality ranged from low to very low across studies.

**Conclusions:**

The SHAPE questionnaire has preliminary support from OMI development and shows promise in some psychometric domains. The NQ lacks pediatric validation. Development of age-appropriate, clinically relevant OMIs is essential for accurate DB assessment in children.

*PROSPERO No*: CRD42024530540.

**Electronic supplementary material:**

The online version of this article (10.1007/s11136-025-04078-8) contains supplementary material, which is available to authorized users.

## Abstract (plain language summary)

Physiotherapists and other healthcare professionals use different tools to check for breathing problems in children. However, we do not know if the tools used can measure change or were made to be used with children. This study looked at the quality of these tools. Two tools were found: the SHAPE questionnaire developed in French and the Nijmegen questionnaire (NQ) originally made for adults. The SHAPE questionnaire worked well when used in French. The NQ was designed for adults and might not be suitable for children, as children’s chests movements during breathing are different. We found that age-appropriate tools are needed to assess children’s breathing problems. This suggests a need to either adapt and test current tools for children or develop new ones that can measure and track breathing.

**Electronic supplementary material:**

The online version of this article (10.1007/s11136-025-04078-8) contains supplementary material, which is available to authorized users.

## Introduction

Dysfunctional breathing (DB) refers to alterations in breathing patterns or asynchronous breathing movement of the chest wall movements that can occur with or without an underlying medical condition [1–3]. However, the definition of DB lacks standardisation, making diagnosis and differentiation from other respiratory diseases challenging [4]. As a result, DB in children is a commonly misdiagnosed condition, as its symptoms closely resemble those of clinical conditions like chronic asthma [1]. The prevalence of DB in children has been reported to range from 5 to 25% of children who are referred to pediatric asthma clinics or identified in schools [5–7]. DB typically presents with symptoms such as dyspnea and hyperventilation and is diagnosed through clinical assessment and a validated questionnaire [8]. Management requires a multidisciplinary approach, including breathing retraining, education, psychological support and self-management strategies [9, 10]. Recognising DB as a treatable trait within the spectrum of respiratory disorders supports personalized asthma care by enabling targeted interventions. Misdiagnosis and inappropriate treatment not only reduce quality of life (QoL) but also lead to unnecessary healthcare costs [11].

Most existing research on DB mainly focuses on adults, with limited data available for the pediatric populations [11, 12]. Children’s respiratory physiology differs from adults with characteristics such as a more rapid breathing rate, increased chest wall compliance, and different respiratory muscle use [13]. Examples of pediatric DB patterns include upper-chest breathing, rapid shallow breathing, and attempts to breathe through an obstructed larynx which can result in clinical symptoms, with or without accompanying hypocapnia [14]. These differences underscore the need for pediatric specific assessment tools, as misdiagnosis and suboptimal treatment can negatively impact QoL [11]. Early and accurate diagnosis is essential for effective management. Despite this, DB assessments in children are rarely conducted and often rely on observational methods or OMIs developed for adults, which lack pediatric validation. We propose using OMIs not only to support clinical diagnosis but also to monitor symptom burden and treatment response over time. This dual role promotes early identification and intervention, aligns with treatable traits, and supports personalised asthma management.

Despite the availability of several adult focused OMIs such as patient reported outcome measures (PROMs), clinician-reported outcome measures (CROMs), and performance-based tests there is a lack of standardised, validated tools for pediatric use. Implementing OMIs in children requires rigorous evaluation of their psychometric properties, including content validity, structural validity, internal consistency, cross-cultural validity, measurement variance, reliability, measurement error, criterion validity, hypothesis testing for construct validity and responsiveness, in line with COnsensus-based Standards for the selection of health Measurement Instruments (COSMIN) guidelines [15]. Although our recent systematic review found that the Nijmegen questionnaire (NQ) meets many COSMIN criteria for adults [12], pediatric specific OMIs remain underdeveloped. Previous reviews have identified OMIs such as NQ, the Hyperventilation Syndrome Ambroise-Pare’(SHAPE) questionnaire, the Self-Evaluation of Breathing Questionnaire (SEBQ), the Breathing Pattern Assessment Tool (BPAT), and other QoL questionnaires for laryngeal obstruction [4, 16], yet without evaluating their psychometric robustness in pediatric populations.

This review aims to support clinicians and researchers by identifying the OMIs appropriate for assessing pediatric DB and determining whether new PROMs need to be developed. By systematically evaluating psychometric properties of current OMIs using COSMIN standards, we aim to inform clinical practice and research, enabling better symptom monitoring and diagnostic precision in children.

## Methods

This systematic review was prospectively registered with PROSPERO [CRD42024530540] and conducted in accordance with the Preferred Reporting Items for Systematic reviews and Meta-Analysis (PRISMA) statement [17, 18]. An experienced librarian supported the development and execution of the search strategy (DY). The review included searches of the EbscoHost CINAHL Ultimate, Cochrane Library, Ovid Excerpta Medica Database (Embase), Ovid EMCare and Ovid Medline databases, covering all records from their inception until October 10, 2024. The search strategy incorporated key terms and subject headings based on a framework that included theoretical constructs (e.g., DB), measurement instruments (e.g., assessment instruments) and outcomes (e.g., validity and reliability) with additional terms related to children. The detailed search strategy is presented in Supplementary Information **S1**. Additionally, we screened the reference lists of included studies and relevant reviews and performed citation tracking of included studies.

### Study selection

To be eligible for inclusion, studies had to use OMIs to assess pediatric DB or evaluate the validity and reliability of a DB related scale (or one of its synonyms) in a pediatric population. PROMs and CROMs developed for related conditions such as bronchial asthma, hyperventilation syndrome (HVS) or chronic obstructive pulmonary disease were also considered if applicable to DB. Only full-text articles published in peer-reviewed journals, in English or translated into English, were included. Translations were conducted using a combination of AI-based tools (such as Copilot and Google Translate) and were subsequently reviewed and confirmed by a French language teacher who is a native speaker to ensure accuracy. Studies were included only if published from 1990 onward, as the term ‘DB’ emerged in that year. Studies were excluded if the OMIs did not specifically assess DB, were laboratory-based, or focused on sleep apnea. Additionally, systematic reviews, commentaries, letters to the editor, and animal studies were not included.

### Data extraction

**Fig. 1:**
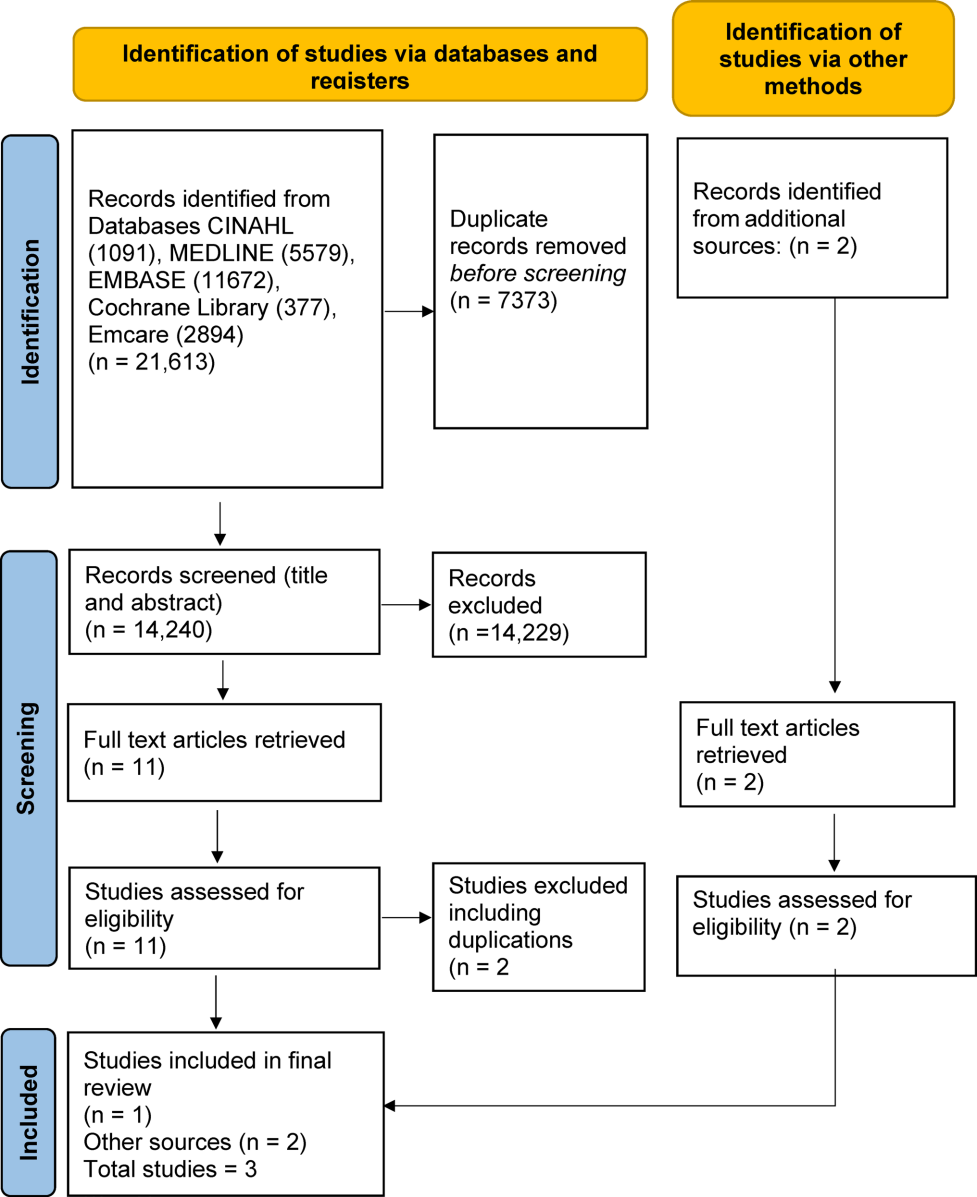
PRISMA flow chart

Two reviewers (VM and CR) independently screened all studies by title and abstract, excluding irrelevant ones. Reference lists of included studies were also searched to identify additional eligible articles. Full texts of potentially relevant studies were retrieved and reviewed by the same two authors (VM and CR). Any disagreements, during the screening or full text review process were resolved through discussion, and if necessary, consultation with a third reviewer. The PRISMA flow diagram outlining this procedure is presented in Fig. 1, following the PRISMA 2020 statement [17].

#### Methodological quality (risk of bias) and quality of results

The review team followed the COSMIN methodology for systematic reviews of PROMs and CROMs to evaluate the psychometric properties of OMIs used in children with DB [19–21].

Two reviewers (VM and CR) independently extracted and assessed data for each psychometric property listed in the COSMIN tool. Methodological quality was rated using the COSMIN risk of bias checklist, applying four-point scale (‘very good’, adequate’, doubtful’, or ‘inadequate’) according to the ‘*worst score counts’* principle, where the lowest rating within a domain determined the overall score [21]. The COSMIN criteria for good measurement properties were then used to rate the results. Definitions of measurement properties and the thresholds used to rate them are summarized in Appendix 1.

The results from development and content validity studies were evaluated using the criteria of relevance, comprehensiveness, and comprehensibility. Based on these criteria, the quality of the results of content validity was graded as sufficient (+), insufficient (−), or indeterminate (?) according to COSMIN standards. For all other measurement properties, the methodological quality was rated similarly, and the results were graded as sufficient (+), insufficient (−), indeterminate (?), or inconsistent (±) based on comparison with COSMIN thresholds [19–21]. Discrepancies between reviewers were resolved through discussion or, if needed, by consulting a third reviewer. For each measurement property, the overall rating was synthesized across studies. If at least 75% of studies agreed on the rating, that rating was assigned. In cases of unexplained inconsistency, the property was rated as inconsistent (±); however, when ≥ 75% of results supported the same conclusion, a majority of rating was applied in line with COSMIN guidance [19–21]. For OMIs evaluated in more than two studies, the full range (from lowest to highest) of ratings for each measurement property was presented to illustrate variability. Finally, a GRADE (Grading of Recommendations Assessment, Development and Evaluation) approach was used to assess the overall quality of evidence for each measurement property, categorized as high moderate, low or very low [19, 20].

#### Patient and public involvement

Although patients and caregivers were not formally involved in the design or conduct of the systematic review, service users and pediatric respiratory specialists were consulted during the broader research process. Input was first sought through an online survey to gauge interest, followed by structured interviews. The semi-structured interviews involved one service user, three respiratory physiotherapists, and two respiratory consultants. Their contributions helped shape the focus of the review by highlighting limitations in existing OMIs in capturing symptoms and supporting communication in clinical care. This feedback underscored the importance of patient reported outcomes in routine practice and informed the direction of the review, which aimed to identify and evaluate existing OMIs for DB in children.

## Results

The search initially produced 21,613 references. After removing duplicates, 14,240 records were included for title and abstract screening. Three full-text articles met the inclusion criteria: one was identified through database searching [22] and two were located via manual screening of reference lists and forward citation searches using Google Scholar [23, 24]. Two of the included articles were in French [23, 24]. One was translated into English using ScienceDirect’s translation feature [23]. The other was translated using Copilot and Google Translate [24]. A French language teacher reviewed the later translation, and VM checked the final English version for accuracy [24]. The data were further checked by a co-author (AV) who was not involved in the initial screening or data extraction.

### Overview of outcome measurement instruments (OMIs)

The search identified two DB related OMIs that had reported psychometric properties, which were: the Nijmegen Questionnaire (NQ) [22] and the Hyperventilation Syndrome Ambroise-Paré (SHAPE) questionnaire [23, 24] (see Table 1). All studies involved participants diagnosed with either DB or HVS.

**Table 1:** Characteristics of outcome measurement instruments (OMIs)

OMIs [Country-language-Year of publication]	No of items recall period	Subscales/scoring	Sample size	Professionals involved	Population studied	Measurement properties examined
Nijmegen questionnaire [France–English; 2022] [22]	16 /Undefined	No subscales / 16 items, Likert scale (0–4); Total score range: 0–64; cut-off 2	50 [G 1: n = 25, female/male: 18/7; G 2: n = 25, female/male: 18/7; n = 25]	2 Medical doctors Physiotherapist Psychologist	G 1: Inappropriate hyperventilation & G 2: DB without hyperventilation	Hypothesis testing for construct validity (Convergent validity) +
Hyperventilation Syndrome Ambroise-Pare Enfant (SHAPE) [France—French; 2008] [24]	Initially proposed 39 (16 respiratory symptoms & 23 non-respiratory symptoms)/ Undefined Finally Proposed 10 (5 respiratory & 5 non-respiratory symptoms)/undefined	2 (Respiratory and Non-respiratory) / Visual Analogue scale (0–100); thresholds ≥ 40 for positive score	85 [G 1: n = 25, G 2:20, G 3: 20, G 4: 20]	Specialized consultants	G 1: Children with HVS alone G 2: Asthma with HVS *Control group* G 3: Children with Asthma without HVS G 4: Presenting with Trauma	PROM development Content validity + Structural validity (Principal component analysis) +
Hyperventilation Syndrome Ambroise-Pare Enfant (SHAPE) [France–French; 2009] [23]	17 (7 respiratory & 10 non-respiratory symptoms)/ Undefined	2 (Respiratory and Non-respiratory)/Visual Analogue scale (0–100) converted to 3-point scale; Major signs score 0-3-6, Minor signs scored 0-1-2; Diagnostic threshold ≥ 25	85 [G 1: n = 25, G 2:20, G 3: 20, G 4: 20]	?	G 1: Children with HVS alone G 2: Asthma with HVS Control group G 3: Children with Asthma without HVS G 4: Presenting with Trauma	PROM development + Content validity + Construct validity (Discriminative validity) + Criterion validity +

G: group; DB: dysfunctional breathing; HVS: hyperventilation syndrome; ^+^ Reviewers inference of the measurement property

The NQ consists of 16 items designed to assess symptoms associated with HVS [22]. Each item is rated on a 5-point Likert scale ranging from 0 (“never”) to 4 (“very often”), yielding a maximum total score of 64 [22]. The items cover a range of symptoms commonly linked to HVS, including respiratory complaints (e.g., shortness of breath), cardiovascular symptoms (e.g., palpitations), gastrointestinal issues (e.g., bloating), and anxiety-related sensations (e.g., dizziness, tingling).

The SHAPE questionnaire, originally developed to identify HVS symptoms in children, underwent two major adaptations [23, 24]. The initial version comprised 39 items, which were later reduced to a 10-item screening tool [24]. In this version, if a child reports at least one of the five respiratory symptoms or five non-respiratory symptoms with a visual analogue scale (VAS) score of ≥ 40, it is considered a positive screening result, indicating the need for further clinical consideration [24]. A more recent adaptation of the SHAPE questionnaire includes 17 items (7 respiratory and 10 non-respiratory), each rated on a 5-point Likert scale ranging from 0 to 4. Higher scores reflect a greater symptom burden, and a score of 25 or above may suggest HVS in children. This version is designed to capture the severity and progression of symptoms over time [23]. The SHAPE questionnaire adopts a symptom-based structure like the NQ encompassing a range of complaints commonly linked to HVS, including respiratory symptoms (e.g., difficulty breathing in, sighing), cardiovascular manifestations (e.g., palpitations), gastrointestinal issues (e.g., abdominal bloating), and anxiety related sensations (e.g., dizziness, tingling).

### Development and content validity studies

A summary of the development process and content validity evidence for the included OMIs is provided in Table 2. Of the two OMIs reviewed, only the SHAPE questionnaire has documented development and content validity specifically for the pediatric population [23, 24]. In line with COSMIN guidelines for evaluating PROM development, the SHAPE questionnaire involved representative pediatric participants during its development to ensure SHAPE reflected the perspective and priorities of the target population [15, 19, 24]. The developmental process followed these steps:

1. Based on existing literature, a preliminary list of clinical signs was compiled including 16 respiratory and 23 non-respiratory symptoms.
2. The initial version was tested on a representative sample comprising individuals diagnosed with HVS and healthy controls.
3. Principal Component Analysis (PCA) was conducted to reduce the number of variables and refine the questionnaire items.

**Table 2:** Evidence synthesis of developmental and content validity of the SHAPE questionnaire using the COSMIN checklist

Outcome measurement Instruments [Country-language; year]	Quality of PROM development	Quality of content validity^+^			Reason for quality rating and additional comments
		Relevance-professionals	Comprehensiveness-professionals	Comprehensibility-patients	
SHAPE Questionnaire (SHAPE-French) [France–French; 2008] [24]	D	NA	NA	NA	‘D’–Only quantitative methods; unclear if development suited construct/population
The study below proposed 17 clinical signs for assessing hyperventilation syndrome based on the signs identified in the above article					
SHAPE Questionnaire (SHAPE-French) [France–French; 2009] [23]	D	NA	NA	NA	‘D’–Only quantitative methods; unclear if development suited construct/population

Quality rating: V = very good; A = adequate; D = doubtful; I = inadequate; Quality score: sufficient (+); insufficient (–); indeterminate (?); inconsistent (±); NA = not applicable; ^+^ Reviewers inference of the measurement property

A subsequent study proposed a revised version of the SHAPE questionnaire with 17 clinical signs (7 respiratory and 10 non-respiratory), and the questionnaire was tested on a representative sample of individuals with HVS [23].

The methodological quality of the SHAPE studies for PROM development was rated as ‘doubtful’ due to the use of inadequate data collection methods for identifying relevant items for a new PROM. Regarding content validity, which we inferred from the studies, it was unclear whether this property was assessed, as there was no mention of asking patients or professionals about the relevance, comprehensiveness, and comprehensibility of the items. The studies were then rated based on PROM development, content validity, and the reviewer’s assessments. The final pooled ratings were ‘indeterminate’ due to the absence of qualitative elements in the content validity evaluations, which we inferred from the included studies. Grading of the evidence was not conducted, as the results were rated as ‘indeterminate’. In contrast, no studies were identified that address the development or content validity of the NQ in pediatric populations (Supplementary Information S2).

### Risk of bias assessment rating of other measurement properties

The evidence synthesis for the measurement properties of the SHAPE questionnaire and the NQ measurement properties is summarized in Table 3 and Supplementary Information S2. For the SHAPE questionnaire, the methodological quality of the study assessing structural validity was rated as *‘inadequate,’* due to missing statistical details such as factor loadings, cross-loading, and explained variance [24]. However, the quality of the structural validity outcome was considered *‘sufficient’,* based on the interpretable and theoretically sound factor structure derived from PCA [24]. In a subsequent study, the criterion validity and discriminative validity of the SHAPE questionnaire were evaluated [23]. Both methodological quality and outcome ratings for these properties were ‘*very good’*, and ‘*sufficient*’, respectively. The SHAPE questionnaire was shown to significantly distinguish between children with and without HVS, supporting its construct validity. An optimal threshold was identified using values of 0, 3, and 6 for major signs and 0, 1, and 2 for minor signs. At this threshold, the instrument achieved an area under the curve (AUC) of 0.934, a specificity of 0.90, and a sensitivity of 0.82, demonstrating strong diagnostic accuracy.

**Table 3:** Methodological quality and rating of psychometric properties in studies involving patients

Outcome measurement Instruments [Country-language; year of publication]	Structural validity*	Internal consistency	Cross-cultural validity/Measurement invariance	Reliability	Measurement error	Criterion validity*	Hypothesis testing for construct validity*	Reason for methodological quality/Additional comments
SHAPE Questionnaire (SHAPE-French) [France–French; 2008] [24]	I (+) PCA: 3 dimensions (respiratory), 3 dimensions (extra-respiratory) Eigenvalue thresholds: ≥ 1.2/ ≥ 1.5	NA	NA	NA	NA	NA	NA	Structural validity*: the reviewers rated the sample size as ‘inadequate’ because it was less than five times the number of items
SHAPE Questionnaire (SHAPE-French) [France–French; 2009] [23]	NA	NA	NA	NA	NA	V (+) Sp = 0.90 & Se = 0.82	V ( +) Discriminative validity: AUC = 0.934	Validity: Reviewers inferred that the questionnaire has the ability to discriminate between HVS and non-HVS
SHAPE–Overall rating (Pooled or summary result)	+	NA	NA	NA	NA	+	+	Results ratings were not pooled because one study looked at structural validity, while the other focused on criterion and discriminative validity. Each was summarized separately
Nijmegen Questionnaire (NQ-French) [France–French; 2022] [22]	NA	NA	NA	NA	NA	NA	I (+) Positive correlation: NQ with dyspnea recorded during HVPT (*p* = 0.010)	Convergent validity*: the reviewers rated the measurement properties of HVPT as ‘inadequate’

Methodological quality rating: V = very good; A = adequate; D = doubtful; I = inadequate; Result score: sufficient (+); insufficient (–); indeterminate (?); AUC: Area under the curve; Sp: specificity; Se: sensitivity; HVPT: hyperventilation provocation test; PCA: principal component analysis; * Reviewers inference of the measurement property

For the NQ, convergent validity was the relevant measurement property assessed. Although the methodological quality of the study was rated as ‘*inadequate*’, the measurement property itself received a ‘*sufficient*’ rating [22]. A significant positive and moderately strong correlation was reported between the NQ and dyspnea recorded during the Hyperventilation Provocation Test (HVPT) with R = 0.42 (*p* = 0.010) at 30% and R = 0.37 (*p* = 0.020) at 40% of predicted maximal ventilation, supporting construct validity through hypothesis testing [22].

### Grading of recommendations assessment, development and evaluation (GRADE)

There is ‘very low’ quality of evidence for sufficient structural validity, based on findings from a single study with ‘inadequate’ methodological quality and an ‘extremely serious’ risk of bias, for the SHAPE questionnaire [24]. There is ‘low’ quality evidence for sufficient criterion validity and construct validity (hypothesis testing), based on a single study with ‘very good’ methodological quality. However, the rating was downgraded due to imprecision, as the results have not yet been replicated, for the SHAPE questionnaire [23].

There is ‘very low’ quality of evidence for sufficient construct validity (convergent validity), with ‘inadequate’ methodological quality, population indirectness, and an ‘extremely serious’ risk of bias, despite a sufficient rating for the measurement property, for the NQ when applied in a pediatric population [22].

## Discussion

This systematic review provided an overview of OMIs used to assess DB and evaluated their psychometric properties in pediatric populations. Among the OMIs identified, the SHAPE questionnaire is the only OMI developed specifically for children, though its psychometric evaluation remains in the early stages.

### SHAPE questionnaire

Developed in French for a pediatric population with DB, the SHAPE questionnaire has undergone initial evaluation for several measurement properties, including content validity, structural validity, criterion validity, and discriminative validity [23, 24]. A weakness in the SHAPE questionnaire was that cognitive interviews, an essential step in OMI development to assess readability and item interpretation, were not conducted during SHAPE’s development [23, 24]. Nevertheless, based on COSMIN guidelines, the SHAPE questionnaire fulfilled the general design standards as the questionnaire was studied in a group representative of the target population for which the PROM was developed. The instrument was developed by identifying key clinical signs (respiratory and non-respiratory) from the literature that distinguish children with HVS from both healthy controls and children with asthma [24]. However, because proper qualitative methods were not used, the overall PROM development quality was rated as ‘doubtful’ and the SHAPE remains in a preliminary stage.

In a subsequent study, additional measurement properties were evaluated [23], with high specificity and sensitivity reported, supporting criterion validity. However, the exact cut-off points and dichotomisation process were not described, limiting interpretability. According to COSMIN, sensitivity and specificity should only be applied to dichotomous outcomes, and the lack of transparency in this process weakens the strength of the conclusions [20]. Overall, both studies contributed preliminary support for the SHAPE questionnaire’s criterion and construct validity (via hypothesis testing) [23, 24]. However, key properties such as reliability, responsiveness, and measurement error were not assessed, highlighting the need for further psychometric evaluation. Without establishing these measurement properties, particularly reliability and measurement error, validity testing may be compromised, as it becomes unclear whether the instrument consistently and accurately measures the intended construct.

### Nijmegen questionnaire (NQ)

The NQ though widely used in adults, presents limitations when applied to pediatric populations. While it includes many measurement properties documented in adult research [12], these cannot be assumed valid in children due to developmental differences in breathing physiology and symptom expression [13]. Additionally, an earlier study evaluating the NQ alongside the hyperventilation test reported a sensitivity and specificity of 56.3%, indicating poor diagnostic accuracy in practice [25]. Although the NQ was included in this review to evaluate convergent validity, the evidence to support its use in children is insufficient. Its language, symptom descriptors, and scoring system may not be appropriate for pediatric populations [22]. This highlights a significant gap in validated OMIs for assessing DB in children. In contrast to adults, where several OMIs are available, research in children remains limited. This is likely due to the complexities involved in pediatric research, ethical considerations, and the variability in symptom presentations across different stages of development. Addressing these challenges is essential for improving diagnostic accuracy and guiding effective treatment strategies in pediatric DB.

The SHAPE questionnaire is promising but requires further development and validation. Greater attention must be paid to ensuring content relevance, language clarity, and symptom descriptor appropriateness for children of different ages and abilities. Evidence suggests that children can reliably describe their symptoms using consistent descriptors [26], indicating that self-reported experiences, if appropriately elicited, are a valid basis for OMI development. For example, children with asthma have been shown to consistently use terms like ‘tight chest’ or ‘hard to breathe’ to describe their symptoms, demonstrating their capacity to articulate relevant experiences [27]. This supports the need for OMIs that are sensitive, reliable, and reflective of children lived experiences, which can in turn facilitate more accurate diagnosis and better targeted interventions. In line with the James Lind Alliance research priorities, future efforts should focus on the development of standardized, age-appropriate OMIs that support monitoring patient progress and evaluating physiotherapy outcomes [28].

Our review highlights the urgent need for validated, developmentally appropriate OMIs for assessing DB in children. While the SHAPE questionnaire offers an early foundation, its current psychometric evaluation is limited. Existing OMIs such as the NQ are not sufficiently adapted or validated for pediatric use. Stakeholder feedback gathered during the broader research process, including the input from pediatric respiratory clinicians and service users, supports the view that current OMIs lack sensitivity across different age groups. In the absence of reliable and standardized tools, DB may continue to be underdiagnosed or misclassified in children. This can hinder effective care, reduce the comparability of research findings, and negatively impact the quality of life for affected individuals. Therefore, developing robust pediatric specific OMIs remains a key priority for advancing both clinical practice and research in this field.

### Limitations

The review has some limitations. First, two of the included articles were originally published in French and translated into English using automated tools and informal translation support, rather than professional translation services. This may have affected the accuracy of technical terminology or context specific nuances, potentially influencing the interpretation of findings. Second, although the reviewers (VM & CR) had no formal training in applying the COSMIN methodology, this was addressed through the involvement of co-author (OLA). Co-author (OLA), provided methodological guidance and supported the assessment of the studies using the COSMIN tool.

## Conclusions

In summary, while the SHAPE questionnaire represents a promising effort to measure DB in children, its current psychometric evaluation is incomplete. To ensure reliable assessment and support clinical decision making, future studies should prioritise psychometric testing of SHAPE or develop a new OMI that addresses the limitations identified in this review. A pediatric specific, psychometrically sound OMI is critical for advancing diagnosis, treatment, and research in this population.

## Electronic supplementary material

Below is the link to the electronic supplementary material.

Supplementary file 1 (XLSX 6 kb)

## Data Availability

The data supporting the results can be found in supplementary information (S1 and S2).

## Appendix

See Table 4.

**Table 4:** Definitions and thresholds of the measurement properties based on COSMIN guidelines [20]

Measurement property	Definition	Threshold for ‘Sufficient rating’
1. Content validity	Degree to which the content of an instrument is an adequate reflection of the construct to be measured	≥ 85% of items of PROM (or subscale) fulfil the criterion
*Internal structure*		
2. Structural validity	The degree to which the scores of a PROM are an adequate reflection of the dimensionality of the construct to be measured	Evidence of good model fit from factor analysis (e.g., CFI/TLI > 0.95, RMSEA < 0.06, SRMR < 0.08), and no violations of unidimensiolity, local independence, or monotonicity
3. Internal consistency	The degree of the interrelatedness among the items	At least low evidence for sufficient structural validity and Cronbach’s alpha(s) ≥ 0.70 for each unidimensional scale or subscale
4. Cross-cultural validity/measurement invariance	The degree to which the performance of the items on a translated or culturally adapted PROM are an adequate reflection of the performance of the items of the original version of the PROM	No important differences found between group factors (such as age, gender, language) in multiple group factor analysis OR no important DIF for group factors (McFadden’s R^2^ < 0.02)
*Remaining measurement properties*		
5. Reliability	The proportion of the total variance in the measurements which is due to ‘true’ differences between patients	ICC or weighted Kappa ≥ 0.70
6. Measurement error	The systematic and random error of a patient’s score that is not attributed to true changes in the construct to be measured	SDC or LoA < MIC
7. Criterion validity	The degree to which the scores of a PROM are an adequate reflection of a ‘gold standard’	Correlation with gold standard ≥ 0.70 OR AUC ≥ 0.70
8. Hypothesis testing for construct validity	The degree to which the scores of a PROM are consistent with hypotheses (with regard to relationships to scores of other instruments, or differences between relevant groups) based on the assumption that the PROM validly measures the construct to be measured	The results of all studies should be taken together, and it should then be decided if 75% of the results are in accordance with the hypotheses
9. Responsiveness	The ability of a PROM to detect change over time in the construct to be measured	Same as above OR AUC ≥ 0.70

AUC: area under the curve; CFA: confirmatory factor analysis; CFI: comparative fit index; DIF: differential item functioning; IRT: item response theory/Rasch methods; ICC: intraclass correlation coefficient; RMSEA: root mean square error of approximation; SRMR: standarised root mean square residual; SDC: smallest detectable change; TLI: tucker-Lewis Index
